# Magnetic separation of peripheral nerve-resident cells underscores key molecular features of human Schwann cells and fibroblasts: an immunochemical and transcriptomics approach

**DOI:** 10.1038/s41598-020-74128-3

**Published:** 2020-10-28

**Authors:** Kaiwen Peng, David Sant, Natalia Andersen, Risset Silvera, Vladimir Camarena, Gonzalo Piñero, Regina Graham, Aisha Khan, Xiao-Ming Xu, Gaofeng Wang, Paula V. Monje

**Affiliations:** 1 Stark Neurosciences Research Institute and Department of Neurological Surgery, Indiana University School of Medicine, Indianapolis, IN USA; 2 The Miami Project To Cure Paralysis, University of Miami Miller School of Medicine, Miami, FL USA; 3 Department of Neurological Surgery, University of Miami Miller School of Medicine, Miami, FL USA; 4 Department of Human Genetics, University of Miami Miller School of Medicine, Miami, FL USA; 5 Interdisciplinary Stem Cell Institute, University of Miami Miller School of Medicine, Miami, FL USA; 6 Division of Spine Surgery, Department of Orthopedics, Nanfang Hospital, Southern Medical University, Guangzhou, China; 7 University of Utah, Salt Lake City, UT USA; 8 Instituto de Investigaciones Bioquímicas de Bahía Blanca (CONICET), Departamento de Biología, Bioquímica y Farmacia, Universidad Nacional del Sur, Bahía Blanca, Argentina; 9 Facultad de Farmacia Y Bioquímica, Departamento de Química Biológica, and CONICET, Instituto de Química Y Fisicoquímica Biológicas (IQUIFIB), Universidad de Buenos Aires, Buenos Aires, Argentina

**Keywords:** Biological models, Genomic analysis, Immunological techniques, Isolation, separation and purification, Cellular neuroscience, Glial biology, Molecular neuroscience, Peripheral nervous system, Regeneration and repair in the nervous system, Biological techniques, Neuroscience, Cell biology, Cell signalling, Senescence

## Abstract

Nerve-derived human Schwann cell (SC) cultures are irreplaceable models for basic and translational research but their use can be limited due to the risk of fibroblast overgrowth. Fibroblasts are an ill-defined population consisting of highly proliferative cells that, contrary to human SCs, do not undergo senescence in culture. We initiated this study by performing an exhaustive immunological and functional characterization of adult nerve-derived human SCs and fibroblasts to reveal their properties and optimize a protocol of magnetic-activated cell sorting (MACS) to separate them effectively both as viable and biologically competent cells. We next used immunofluorescence microscopy imaging, flow cytometry analysis and next generation RNA sequencing (RNA-seq) to unambiguously characterize the post-MACS cell products. High resolution transcriptome profiling revealed the identity of key lineage-specific transcripts and the clearly distinct neural crest and mesenchymal origin of human SCs and fibroblasts, respectively. Our analysis underscored a progenitor- or stem cell-like molecular phenotype in SCs and fibroblasts and the heterogeneity of the fibroblast populations. In addition, pathway analysis of RNA-seq data highlighted putative bidirectional networks of fibroblast-to-SC signaling that predict a complementary, yet seemingly independent contribution of SCs and fibroblasts to nerve regeneration. In sum, combining MACS with immunochemical and transcriptomics approaches provides an ideal workflow to exhaustively assess the identity, the stage of differentiation and functional features of highly purified cells from human peripheral nerve tissues.

## Introduction

Primary cell cultures are commonly jeopardized by the outgrowth of cells that are able to divide at higher rates than the desired cell type. Contaminating cells often consist of fibroblasts, the most prevalent cell type in connective tissue, whose primary function is to produce and maintain the structure of the extracellular matrix (ECM). Primary Schwann cell (SC) cultures are no exception, as the elaborate connective tissue layers within the epineurium, perineurium and endoneurium contain abundant fibroblasts with potential to proliferate in the SC cultures. The risk of fibroblast overpopulation is a major hurdle in establishing and maintaining SC cultures from adult human peripheral nervous system (PNS) tissues^1,2^, especially in late passage cultures^3^ and co-cultures with neuronal cells^2^. It has been shown that broad-spectrum anti-mitotic drugs, such as cytosine arabinoside^4^, and agents that elevate intracellular cAMP levels, such as forskolin and cholera toxin, can be used to control the number of fibroblasts^5,6^ . However, fibroblast elimination in cultures of human origin is rarely achieved. Despite the fact that contaminating cells are often referred to as fibroblasts, their origin and special features are still uncertain. New research in rodent models have suggested cooperation between SCs and fibroblasts in supporting nerve growth after injury^7,8^ but no evidence so far indicates that a similar case occurs in human cells.

Magnetic-activated cell sorting (MACS) provides an advantage to rapidly (i.e., within 2 h) purify rat SCs at high efficiency without killing the fibroblasts^9^. However, we found that direct translation of purification methods from rats to humans was mostly ineffective^10,11^ due to species-specific variations in the antigenic properties of the cells and other aspects of their growth control^12^. Consequently, our primary goal was to perform a phenotypic and functional analysis of peripheral nerve-derived human SC cultures to reveal the prevalent cell types in the populations and ascertain the most effective strategy for magnetic cell labeling, separation by MACS and characterization of the post-MACS cell products. Our next goal was to analyze the purified SC populations and establish a comparison to the contaminating cells (herein referred to as fibroblasts generically) to ascertain their respective origins and gene expression profiles. We began this study by investigating antibody combinations able to discern SCs from fibroblasts and ensure their effective purification. This analysis led to the identification of p75^NGFR^, the low affinity neurotrophin receptor, as the most reliable and stable cell surface marker to distinguish human SCs and fibroblasts via immunofluorescence microscopy and flow cytometry analysis using either live or fixed cells. Immuno-tagging with human-specific p75^NGFR^ antibodies led to the recovery of pure SC populations even when the starting preparations contained a predominance of fibroblasts. Ultimately, we used next generation whole transcriptome profiling or RNA-seq to unequivocally characterize the MACS-purified cells. The RNA-seq profiles of human SCs and fibroblasts revealed their non-overlapping molecular signatures underlying their respective neural crest and mesenchymal origins. Strikingly, both SCs and fibroblasts displayed gene expression profiles consistent with progenitor cells capable to promote nerve tissue regeneration though independent mechanisms involving cell–cell signaling via direct membrane contact, secreted soluble factors, and interactions with the ECM.

**Figure 1:**
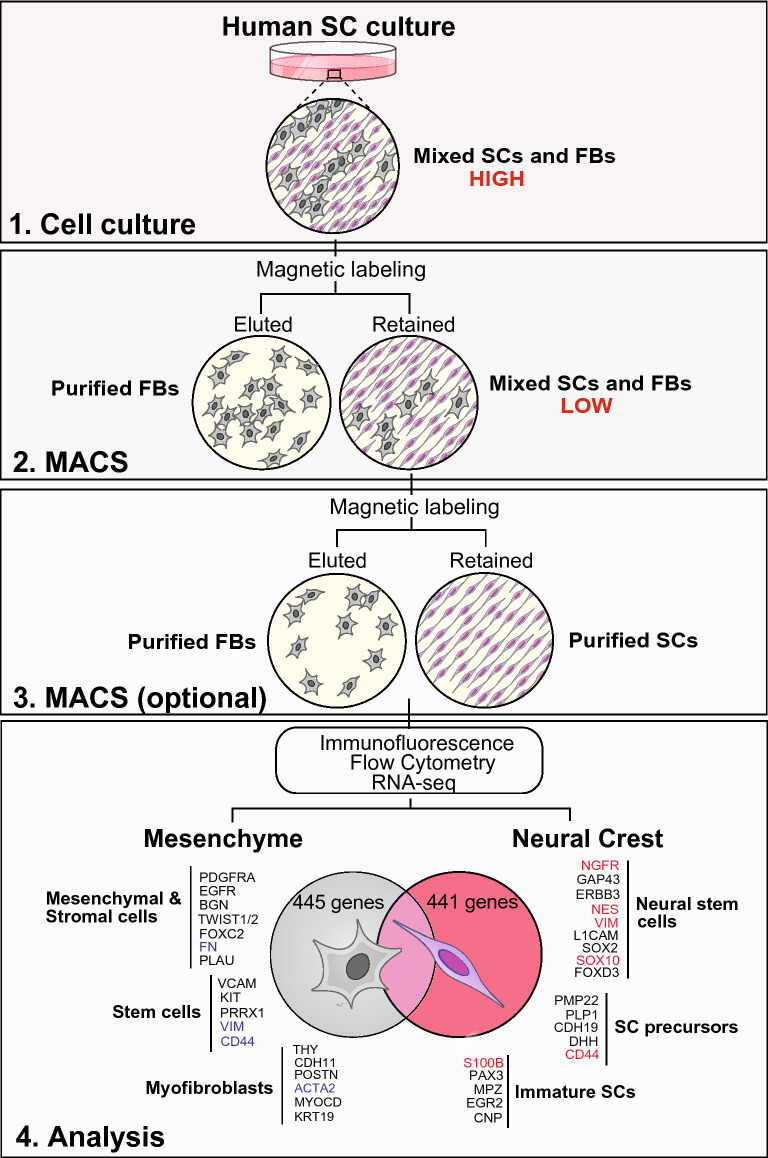
An optimized workflow to efficiently purify and characterize SCs and fibroblasts from adult human peripheral nerve tissue. This protocol uses heterogeneous human cultures containing cells identifiable as SCs and fibroblasts as evidenced by expression of p75^NGFR^, a SC (neural crest)-specific receptor. MACS of p75-labelled cells allowed the fast recovery of SCs and fibroblasts retaining their viability and biological activity. The rounds of MACS required to achieve > 95% SC purity was dependent on the degree of fibroblast growth (high or low, as indicated). Immunofluorescence microscopy, flow cytometry and transcriptome (RNA-seq) analysis were used to confirm the identity and purity of the MACS-purified cells. Some key lineage- and stage-specific genes revealed by RNAseq (bottom panel) emphasized the independent origin of human SCs and fibroblasts, their relative immaturity, and the heterogeneity of the fibroblast populations. The genes highlighted in color were confirmed by immunological methods.

To conclude, we herein present a convenient and adaptable workflow for the culture, purification and analysis of human peripheral nerve-resident cell types (Fig. 1) that was validated using > 10 different cultures. In addition, we present an in-depth analysis of purified SCs and fibroblasts of human origin, which have been understudied when compared to cells from experimental animals^13^. Our workflow can suit the needs of assorted basic and translational projects in which understanding human SC function, or using the cells themselves as a cell product, has relevance to investigations on peripheral nerve development, therapy, or neurodegenerative disease.

## Materials and methods

### Cell culture materials, antibodies and hybridoma cell lines

Cell culture grade water, high-glucose Dulbecco’s Modified Eagle’s medium (DMEM), Iscove’s Modified Dulbecco’s Medium (IMDM), Hanks Balanced Salt Solution (HBSS, calcium- and magnesium-free), Dulbecco's Phosphate-Buffered Saline (DPBS), 0.5% trypsin–EDTA, 100X Glutamax Supplement, 10,000 U/mL penicillin–streptomycin and 50 mg/mL gentamycin were provided by ThermoFisher (Waltham, MA). Fetal bovine serum (FBS) was provided by HyClone-GE Healthcare (Logan, UT). Recombinant heregulin-β1177-244 (herein described as neuregulin) and forskolin were furnished by PreproTech (Rocky Hill,NJ) and Sigma-Aldrich (St. Louis, MO), respectively. Poly-L-lysine (PLL) and mouse laminin (1 mg/mL) provided by Sigma-Aldrich (St. Louis, MO) were used to coat cell-culture treated 10-cm dishes, 6- and 24-well plates (Corning). Primary antibodies used for immunostaining were obtained as follows: anti-S100β (rabbit polyclonal, Cat # Z0311, DAKO Carpinteria, CA); anti-fibronectin (mouse monoclonal, Cat # sc-8422, Santa Cruz); anti-smooth muscle actin or SMA (Cat # MS113-PO, ThermoFisher); anti-vimentin (rabbit monoclonal, Cat # D21H3, Cell Signaling, Danvers, MA); anti-CD44 (mouse monoclonal, Cat # 156-3C11, Cell Signaling), anti-Sox10 (rabbit monoclonal, Cat # ab155279, Abcam), and anti-nestin (Cat # ab6142, Abcam). Fluorescent antibodies used for flow cytometry were the following: APC-conjugated anti-human CD271/p75^NGFR^ (Clone ME20.4, Cat # 345108), and FITC-conjugated anti-human CD90/Thy-1 (Clone 5E10, Cat # 328108) both from Biolegend (San Diego, CA). The hybridoma cell lines clones HB-8737 (primate/human-specific, monoclonal anti-p75^NGFR^) and HFN36.3 (human-specific fibronectin) were acquired from the American Type Culture Collection (ATCC, Manassas, VA). O4 hybridoma cells were a gift of Dr. Mellita Schachner to Dr. Patrick Wood. Alexa Fluor 488, 546 and 647-conjugated antibodies in the form of goat anti-rabbit IgG (Cat # A-11035), goat anti-mouse IgG (Cat # A-11001) and goat anti-mouse IgM (Cat # A21042) were supplied by ThermoFisher. The IgG magnetic microbeads (Cat # 130-048-401) and the Anti-Fibroblast MicroBeads Miltenyi (Cat #130-050-601) were provided by Miltenyi Biotec GmbH (Bergisch Gladbach, Germany).

### Preparation of PLL- and laminin-coated surfaces

Plates were sequentially coated with PLL and mouse laminin to allow for a strong adhesion of the human SCs. A 100X PLL stock solution (2%) was prepared by dissolving the PLL powder in borax/boric acid buffer (1.9% sodium tetraborate and 1.2% boric acid), followed by sterile filtering. From this, a working solution of PLL was prepared in cell culture grade water and used as coating solution to completely cover the surface at a ratio of 1 mL per 25 cm^2^. After a 1-h incubation at room temperature (RT), the PLL-coated substrate was rinsed with cell culture grade water and allowed to dry completely prior to use or storage at 4 °C. The dishes were subsequently coated for at least 1 h with a laminin solution prepared in chilled DPBS at a ratio of 0.7–1 µg of laminin per cm^2^ of coated surface. The laminin solution was removed immediately before seeding the cells with the precaution of not letting the laminin substrate dry at any time. Of note, the adhesion of human SCs to freshly coated laminin plates was not changed when provided in the range of concentration specified above. Laminin was used in the lower range for routine subculture and re-plating in multiwell dishes but a ~ 30% excess of laminin was preferred for cell plating after MACS to enhance the survival of cells subjected to stress conditions. Cells were seeded in 10-cm dishes for routine expansion and MACS separation, 24-well plates for fluorescence microscopy, and 6-well plates for flow cytometry and RNA-seq analysis.

### Culture and expansion of primary human SCs from cryopreserved stocks

Primary human SC cultures were prepared and expanded as described in our previous studies^14,15^ by using methods analogous to those used in clinical trials^10^. The cultures were obtained from postmortem human peripheral nerves made available as deidentified (coded) biospecimens by the National Disease Research Interchange (NDRI) and the Life Alliance Organ Recovery Agency (LAORA) of the University of Miami Miller School of Medicine to the laboratories of Paula Monje and Patrick Wood at The Miami Project to Cure Paralysis, respectively, as described in our previous studies^15,16^. NDRI and LAORA strictly adhere to government regulations and guidelines regarding donor authorization and confidentiality, and ensure that informed consent is obtained from the donors (or the next-of-kin thereof) for research use. In vitro experimentation with human tissues and cells in this project was approved and deemed to constitute non-human subjects research by the Human Subjects Research Offices of the University of Miami and Indiana University. Procedures were further reviewed and approved by the Institutional Biosafety Committee of Indiana University. All experiments were done using banked cell cultures obtained from non-pathological nerve tissues from males and females, ages 10 to 66, that tested negative for blood borne viruses. Cryopreservation of cell stocks was done in medium consisting of dimethyl sulfoxide (DMSO) and FBS at a ratio of 1:9. Human SCs from the nerve roots comprising the cauda equine were used in experimentation unless otherwise noted. Experiments used early passage SC cultures usually collected after one to three rounds of subculture albeit some exceptions, as indicated in the figure legends. The cultures were selected according to their initial content of fibroblasts irrespective of the passage number or donor-specific characteristics such as gender and age.

Cryovials of human SCs were quickly thawed at 37 °C and re-suspended in 10–15 mL of DMEM containing 10% FBS (heat-inactivated *in house*) prior to collection by centrifugation and plating directly onto PLL-/laminin-coated 10-cm dishes at a density of 1–2 × 10^6^ cells /dish. Cells were quantified and tested for viability using the Trypan blue exclusion assay. Cells were cultured in mitogens-supplemented growth media consisting of high-glucose DMEM, 10% FBS, 1% GlutaMAX, 1% penicillin–streptomycin, 10 nM neuregulin and 2 µM forskolin. This media formulation is referred here as ‘SC growth media or mitogens’ in the text and figures. The cells were maintained in a 37 °C incubator set to 8–9% CO_2_ for optimal growth. The cultures were observed under a phase contrast microscope to confirm cell adhesion to the substrate within 2–3 h of seeding. Regular media changes were performed until the cells reached confluency. For passaging, the cells were dislodged from their dishes to obtain a single cell suspension via enzymatic treatment with trypsin/EDTA prepared in calcium- and magnesium-free HBSS. The enzymatic dissociation was monitored by phase contrast microscopy to ensure that a single cell suspension was obtained without overexposing the cells to trypsin. The trypsinization step was interrupted by addition of DMEM containing 10% FBS followed by low speed centrifugation. Cells to be used in MACS were plated in SC growth media at an initial density of 10^6^ cells per 10-cm dish and cultured until confluency. Experiments that used unpurified SC cultures were carried out in mitogens-supplemented medium.

### Fibroblast cultures obtained via differential adhesion to plastic dishes

Prior to establishing MACS protocols, a simple method of differential adhesion was implemented to obtain purified fibroblast cultures for routine experimentation. For this, a single cell suspension of human SC cultures containing fibroblasts was plated onto non-coated 10-cm cell culture dishes and incubated at RT for 15 min to allow the adhesion of fibroblasts but not SCs. The cultures were washed with L15 medium to remove floating cells and the remaining cells (adherent) were expanded in DMEM containing 10% FBS without added mitogens (in uncoated dishes) up until confluency. These cultures were passaged twice in FBS-only medium and banked by cryopreservation^17^. Cells obtained by this method exhibited an S100β negative, p75^NGFR^ negative, fibronectin positive phenotype. This protocol was useful to reduce the number of fibroblasts in SC cultures that already had a low proportion of contaminating cells (< 20%) but was ineffective to purify massively contaminated cultures.

### Culture of hybridoma cell lines and preparation of conditioned medium

Monoclonal antibodies against p75^NGFR^, O4 and fibronectin were produced *in house,* stored in aliquots at − 80 °C and used in the form of undiluted hybridoma culture supernatant in all cell labeling experiments. The hybridoma cells were plated and expanded as suspension cultures in medium consisting of IMDM supplemented with 10% FBS, 1% GlutaMAX, 1% penicillin/streptomycin and 0.1% gentamycin. These cells were sub-cultured at a 1:4 ratio in T75 flasks. The culture supernatant was collected by low speed centrifugation after each round of expansion. The time of collection was determined when it was visually appreciated that the medium turned yellow in color^11^. Every batch of hybridoma culture supernatant (herein referred to as ‘conditioned medium’) was tested for its labeling activity and specificity using positive control cells. Adherent human SCs and fibroblasts were used to validate the quality of p75^NGFR^, O4 and fibronectin antibodies.

### Live cell immuno-labeling and MACS

For MACS, human SCs were expanded up until at least 10^7^ cells were available per donor or batch. Aliquots containing up to 2 × 10^7^ cells were incubated with 5 mL of undiluted p75^NGFR^ conditioned media for 20 min at 4 °C, with agitation every 5 min to maintain the cells in suspension. The cells were collected by low speed centrifugation, washed with DMEM containing 10% FBS and ultimately resuspended in 80 µL of sorting buffer (0.5% BSA, 2 mM EDTA prepared in D-PBS, pH 7.2) containing 20 µL of anti-IgG antibodies conjugated to magnetic microbeads. Incubation was carried out for 20 min at 4 °C before addition of 5 mL of DMEM containing 10% FBS. Removal of unbound antibodies was carried out by low speed centrifugation. The labeled cells were resuspended in BSA sorting buffer without EDTA prior to applying them to the column/s, following the manufacturer’s instructions (Miltenyi Biotech). Three washes of the column with BSA sorting buffer were performed to elute the cells that did not express p75^NGFR^ (eluted fraction). Subsequently, the columns were detached and the p75^NGFR^ positive cells (retained fraction) were released in 1 mL of sorting buffer after applying positive pressure with a plunger as suggested by the manufacturer. Both the eluted and retained cells were immediately pelleted, re-suspended in DMEM containing 10% FBS, counted and tested for viability. A representative sample of unpurified cells (herein referred to as the ‘total fraction’) was allocated separately from the onset and used as control for quantitative analysis by immunofluorescence microscopy and flow cytometry. The SC-enriched fractions were plated in mitogens medium whereas the fibroblast-enriched fractions were plated in FBS-only supplemented medium to maximize the health and proliferation of each population right after MACS. Importantly, the expression of the markers used for cell type identification via immunostaining were visually unchanged when the fibroblasts were placed in FBS-only medium.

Both MS or Large-Cell columns (Miltenyi) were tested with good results. While both column types rendered purities > 95% SCs, separation by means of Large-Cell columns allowed for a superior yield (i.e. the cells recovered in the eluted and retained fractions combined with respect to the total number of cells passed through the column) despite a proportion of SCs eluted together with the fibroblasts. MS columns were preferred to obtain high purities in both the eluted and retained fractions despite the lower speed of separation and consequent loss in total cell yields. Scalability to larger cell numbers (i.e. > 2 × 10^7^ cells) was rarely needed but was possible by adjusting the volumes of primary and secondary antibodies, and using additional columns as dictated by the number of cells. Technical adjustments to this protocol for an increased speed of separation, enhanced purity and/or yields can be found in our recent publication^11^.

### Determination of cell counts, viability and proliferation

Cells in suspension were stained with a 0.2% solution of Trypan blue and subjected to quantification and vitality assessment using a BioRad TC20 automated cell counter. DNA synthesis was determined by utilizing the Click-iT Plus EdU Alexa Fluor 594 Proliferation Assay Kit (Thermo-Fisher) that uses EdU (5-ethynyl-2′-deoxyuridine) added to the culture medium to label the DNA of actively dividing cells. A reaction with a fluorescent azide allows the visualization of EdU-labelled DNA in dividing nuclei by fluorescence microscopy. The EdU labeling reagent was added to the culture medium of adherent cells at a final concentration of 1–2 µM. It was added 18–24 h after seeding the cells and maintained in the culture medium up until fixation with 4% paraformaldehyde in PBS. The EdU reaction was performed according to the manufacturer’s instructions 48 h after plating. The cells were counterstained with Hoechst 33342 prior to fluorescence microscopy imaging.

[^3^H]-thymidine incorporation assays used sub-confluent cultures of human nerve-resident fibroblasts obtained via selective adhesion to plastic^14^. The fibroblasts were plated on PLL-laminin-coated 24-well dishes (50,000 cells/well) and incubated for 1 d in HEPES-buffered DMEM containing 1% FBS to lower the proliferation rate prior to addition of [^3^H]-thymidine (0.25 µCi/ml) under the conditions described in the figure legends. Incorporated tritium into the cells was determined by liquid scintillation counting typically 72 h post-stimulation. Neuregulin (10 nM), forskolin (2 µM), and FBS (10%) were used alone and in combination to gauge the responsiveness of fibroblasts to the chemical factors used routinely in the formulation of human SC growth media^16^. Other factors used as potential mitogens were the following: db-cAMP (100 µM), PDGF-BB (20 ng/mL), IGF-1 (50 ng/mL), FGF-2 (20 ng/mL), EGF1 (20 ng/ml), and TGFβ (20 ng/mL).

### Identification of senescent cells

A cytochemical assay that uses the chromogenic compound X-gal (5-bromo-4-chloro-3-indoyl β-D-galactopyranoside) as substrate for the pH-dependent β-galactosidase (SA-βGAL) was used to determine senescence in fixed cells, according to the manufacturer’s instructions (Cell Signaling). The cleavage of X-Gal by β-galactosidase at pH 6 renders an insoluble intracellular blue precipitate that allows the visual discrimination of senescent cells. The percentage of stained cells, as seen by bright field microscopy, was determined with respect to the total number of cells, as revealed by fluorescent nuclear staining using DAPI. In some experiments, cells were immunostained with anti-p75^NGFR^ antibodies to discriminate human SCs from fibroblasts.

### Immunofluorescence microscopy

Following cell sorting, replicates of the total, eluted and retained fractions were seeded on PLL-/laminin-coated multi-well dishes to assess cell purity via immunofluorescence microscopy imaging. The MACS-purified cells were allowed to adhere and proliferate for 48 h in SC growth medium. The cells were sequentially fixed with 4% paraformaldehyde and cold methanol (− 20 °C) to allow co-staining with antibodies targeting membrane (e.g. p75^NGFR^, and CD44) and intracellular antigens (e.g. S100β, vimentin, SMA, Sox10, and nestin). Cells were blocked with 5% normal goat serum in PBS prior to addition of the antibody combinations described in the figure legends. After overnight incubation at 4 °C, the cells were washed with PBS and incubated with fluorescent secondary antibodies (1:300) prepared in 5% normal goal serum containing DAPI. Incubation with secondary antibodies was carried out for 1 h at RT, shielded from light. Following staining, the cells were post-fixed, mounted and observed under an Olympus 1X70 inverted fluorescence microscope. Cells were assessed for the positive or negative expression of p75^NGFR^, S100β and other markers in reference to total nuclear staining (DAPI). Large surface areas were scanned via High Content Screening Automated Fluorescent Microscopy (Thermo Scientific Cellomics Arrayscan Vti High Content System Reader, Version 6.6.2.0.). Images of the green (488 nm), red (546 nm) and UV (DAPI) fluorescence shown in the figures were taken from the central area of each well. At least 36 microscopic fields taken at 10× were used for quantification.

### Flow cytometry analysis

Viable cells in suspension were analyzed for the expression of SC- and fibroblast-associated markers by flow cytometry. Each sample consisted of 10^6^ cells comprising the total (unpurified), eluted and retained fractions. Cells were washed with flow cytometry buffer consisting of 1% BSA and 0.1% sodium azide prepared in PBS prior to aliquoting them into equal samples for live cell labeling using fluorescent antibodies against extracellular antigens specifically expressed in human SCs (APC-CD271) and fibroblasts (FITC-CD90), respectively. Cells were labeled with 5 µL of each fluorescent antibody per million cells in 100 µL flow cytometry buffer, as recommended by the manufacturer. Incubation was carried out for 0.5–1 h at RT protected from light. Stained cells were washed twice with flow cytometry buffer, fixed with 4% paraformaldehyde prepared in PBS for 15 min, washed again with flow cytometry buffer, and pelleted by centrifugation. The cells were resuspended in 300 µL of flow cytometry buffer and analyzed within 24 h of staining. At least 10^4^ events per condition were acquired using a Becton–Dickinson CytoFLEX flow cytometer (San Jose, CA, USA). Compensation controls were prepared with both unstained and single-stained samples. Quantification was performed using FlowJo v10.0.8 software (FlowJo, Ashland, OR, USA) after excluding doublets and dead cells.

### RNA isolation, sequencing by RNA-seq and bioinformatics analysis

For RNAseq, we used MACS-purified human SCs and fibroblasts derived from a typical passage-1 culture from a 51 year-old donor. The SCs and fibroblasts were plated for 2 days in mitogens and FBS-only supplemented media, respectively, before collection as a single cell suspension. The cells were allowed to recover after MACS but they were not subjected to subculture. A total of 2 × 10^6^ cells from the retained and eluted fractions were collected by trypsinization, pelleted by centrifugation and stored at − 80 °C for RNA isolation. RNA extraction and DNAase treatment was conducted using the RNeasy Mini Kit (Qiagen, Valencia, CA) following the manufacturer’s instructions. The quality of the RNA was deemed suitable for sequencing on the basis of RNA integrity numbers above 9 as per the BioAnalyzer 2000 (Agilent, Santa Clara, CA). A total of 560 ng of RNA was used for sequencing. RNA-seq was carried out at the Sequencing Core of the John P. Hussman Institute of Human Genomics (University of Miami) using the TruSeq Stranded Total RNA Library Prep Kit (Illumina, San Diego, CA). Briefly, after ribosomal RNA (rRNA) was depleted, sequencing libraries were ligated with standard Illumina adaptors and subsequently sequenced on a Hiseq2000 sequencing system (125 bp paired-end reads, 4 samples per lane; Illumina, San Diego, CA, USA).

Raw sequencing reads were aligned to the reference genome (*Homo sapiens*) and quantified using STAR software. Statistically significant differential expression for each gene was assessed using two software packages: edgeR and DESeq2, as described recently^14^. Density plots were generated and a limit of sensitivity was found to be 2.5 read counts per million (RCPM)^18^. To reduce false positives, only transcripts above 2.5 RCPM in at least one cell type and an adjusted P-value (false discovery rate, FDR) of 0.05 by both DESeq2 and edgeR were considered differential between sample types. Differential transcripts were visualized in the form of a volcano plot using scatterplots in R (https://www.R-project.org). The genes present only in SCs were determined by selecting the transcripts exhibiting a minimum of tenfold greater expression in SCs than in fibroblasts if the read count in fibroblasts was negligible (< 1 RCPM). If the RCPM in fibroblasts was not negligible (> 1 RCPM), a minimum of 50-fold change in expression was used as the cutoff for genes expressed only in SCs to account for up to 2% contaminating fibroblasts in the SC cultures. A similar method was used to determine the genes expressed only in fibroblasts. Pathway analysis was conducted on lists of genes expressed only in one cell type using enrichR^19^. Information from the following databases was used to construct all presented bar graphs and Venn diagrams: Jensen Tissues, Reactome and Gene Ontologies (GO) Biological Processes. Gene Set Enrichment Analysis (GSEA) was used to confirm positive results of pathway analysis and generate enrichment plots^20,21^. Additional technical and scientific information was obtained from GeneCards (https://www.genecards.org/). Annotated names were used for the identification of gene transcripts in the figures. Only the most relevant isoforms/variants in each gene group were selected for presentation. Selection was based on their relative levels of expression (RCPMs or fold induction) as well as their known relevance to the function of the cell types analyzed.

### Statistical analysis

Statistical analysis by means of the Student’s t-Test or One-Way ANOVA was performed using Sigma Plot 12.0 (Systat Software, San Jose, CA). For multiple comparisons, Bonferroni correction was used. Experimental data was expressed as the mean ± standard deviation (SD) of triplicate samples from each experimental condition. Values of significant correlations were indicated in the table and figures according to their degree of significance (* p < 0.05, * * p < 0.01 and *** p < 0.001). Statistical significance was accepted for p < 0.05.

## Results

### Peripheral nerve-derived human fibroblasts are non-senescent proliferative cells

**Figure 2:**
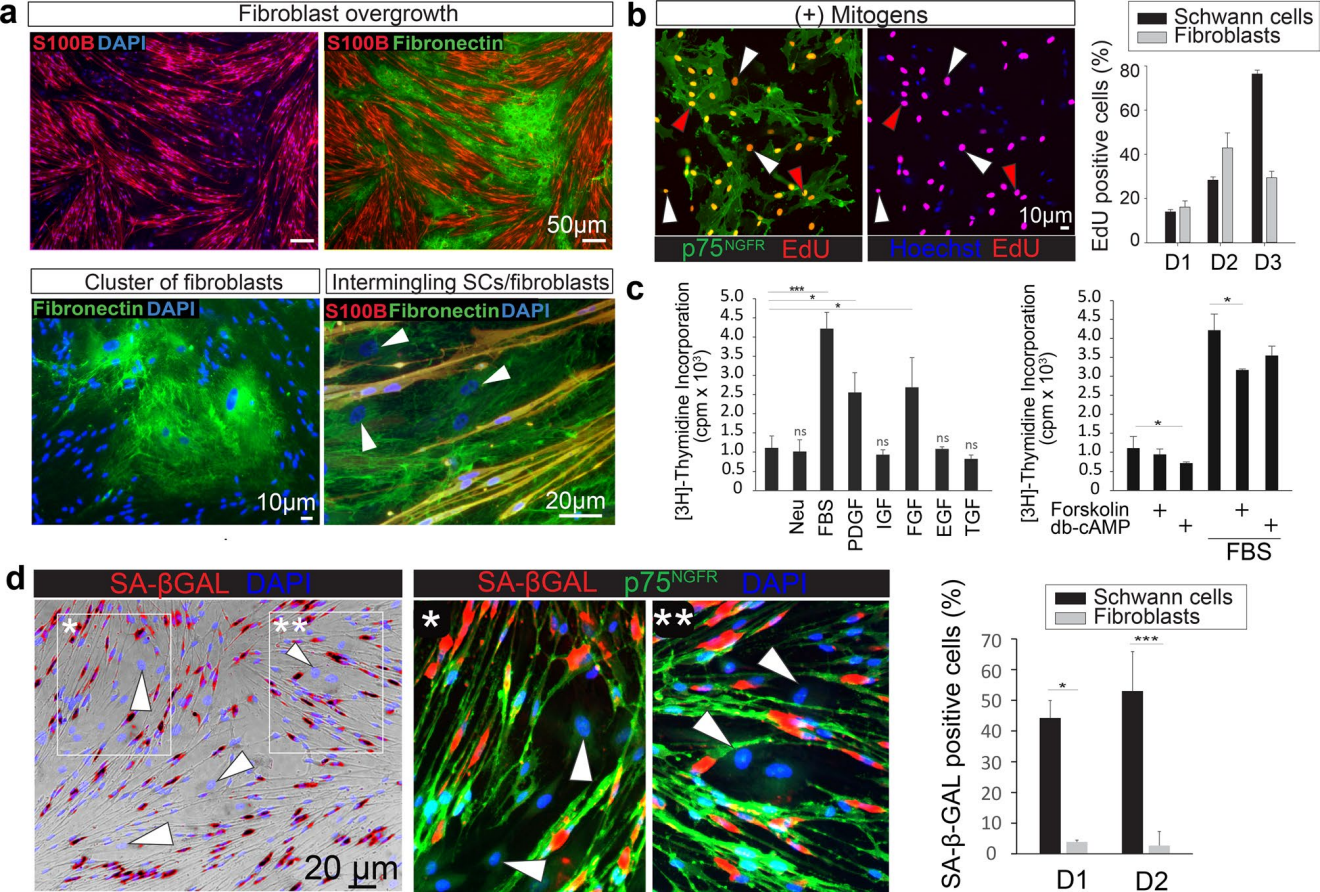
Characterization of adult peripheral nerve-derived human SCs and fibroblasts. (a) Discrimination of SCs and fibroblasts via double immunolabeling with S100β and fibronectin antibodies showing strong cytoplasmic expression of S100β in the SCs and extracellular location of fibronectin in the fibroblasts. Images taken at low (upper panels) and high magnification (lower panels) are provided to illustrate the impact of fibroblast overgrowth in a typical human SC culture (passage-2, 18 year old, male) deprived of mitogenic factors for 10 days. SCs in confluent cultures have a tendency to display an elongated, spindle-shaped phenotype. Although morphological differences can aid in SC versus fibroblast identification, immunostaining is needed for clear discrimination, especially in those areas where the SCs intermingle with the fibroblasts (**a**, lower right panel). (**b**) Fibroblast proliferation in human SC cultures. SC cultures were maintained in SC growth medium (standard conditions) and allowed to incorporate EdU for 3 days before fixation and detection of EdU in the nucleus (red) of SCs (red arrowheads) and fibroblasts (white arrowheads), as determined by p75^NGFR^ immunostaining (green). Quantitative data from 3 independent early passage SC cultures is shown to illustrate the variability in the extent of fibroblast proliferation under optimal conditions for SC growth. Donors were identified as D1 (18 year old, male), D2 (10 year old, female) and D3 (51 year old, male). (**c**) Mitogenic effect of purified growth factors and serum in human fibroblasts obtained by differential adhesion to plastic. Fibroblasts were plated in multiwell dishes to assess the incorporation of [^3^H]-thymidine in the absence (control) and presence of the indicated mitogenic factors (see Methods). Statistical significance was obtained from a One-Way ANOVA. Experimental treatments showing non-significant differences (ns) with respect to the control (no growth factors added, left bars) are indicated. (**d**) Lack of senescence in contaminating human fibroblasts. Late passage cultures (passage-4) enriched in senescent human SCs were used to determine SA-βGAL activity (shown in red as artificial color). Two areas (insets) were selected to depict the distribution of SA-βGAL activity (red) in p75^NGFR^ positive cells (green). Quantitative data and level of significance (T-test) from 2 independent cultures (D1, 18 year old, male, and D2, 51 year old, male) are shown on the right panel. DAPI or Hoechst (blue) was used to label cell nuclei in these and all subsequent fluorescence microscopy images.

SC cultures are usually heterogeneous and contain non-glial cells in different proportions. Fibroblasts are a major component of the contaminating populations (Fig. 2a) but other cell types such as immune cells, stem cells and pericytes can also prevail in cultures of human origin. The non-glial cells can effectively adhere to uncoated, plastic dishes and this differential attribute can be exploited to reduce the impact of fibroblast contamination in SC cultures^22^. Fibroblasts are rapidly proliferating cells that respond to a broad range of growth factors (Fig. 2b–c). This stays in stark contrast to SCs, which are highly selective for, and also strictly dependent on, the addition of neuregulin and cAMP-inducing agents for a maximal rate of growth^5,14,15^. The fibroblasts express ErbB2 but as opposed to the human SCs, they do not express the ligand binding partner of the ErbB co-receptor, ErbB3^23,24^ (Fig. 5b), and fail to proliferate when stimulated with neuregulin (Fig. 2c). Yet, the fibroblasts respond to PDGFBB, FGF-2 and undefined factors present in serum with an increase in cell division (Fig. 2c). Forskolin and other inducers of cAMP (e.g., the membrane permeable analog db-cAMP) moderately reduce the serum (FBS)- and growth factor (PDGFBB and FGF-2)-induced proliferation of fibroblasts (Fig. 2c, and data not shown). This response contrasts sharply with the cAMP-driven synergistic enhancement of neuregulin-induced proliferation typical of human SCs^5,15,16^.

Nevertheless, the addition of cAMP-stimulating agents was only partially effective in preventing fibroblast proliferation, as they continue to divide well even in forskolin-supplemented medium (Fig. 2b–c). Indeed, the simple removal of mitogenic factors (neuregulin/forskolin) can be sufficient to allow them to expand rapidly and overtake the cultures. The magnitude of fibroblast overgrowth is exemplified in Fig. 2a, which shows a panoramic view of an initially > 90% pure human SC culture that was maintained in FBS-supplemented, neuregulin- and forskolin-free medium for 10 days. In these cultures, the fibroblasts developed within densely populated clusters that segregate from the SC bundles. The distinct pattern of growth was revealed by co-immunostaining with antibodies against fibronectin, a major component of the nerve’s ECM, and S100β, a calcium-binding protein widely used as SC marker in human peripheral nerve cultures^25^.

Human nerve-resident fibroblasts can divide at similar or even higher rates than the human SCs in SC growth medium though variability is observed according to the donor (Fig. 2b) and possibly other variables. Strikingly, most non-glial cells failed to undergo senescence under conditions that allowed human SCs to become incompetent to proliferate likely due to senescence, as evidenced by SA-βGal activity assays (Fig. 2d). This is one main reason why, if not eliminated in due time, the fibroblasts can overwhelm the human SC cultures in a few rounds of subculture.

In closing, the human fibroblasts exhibit a competitive advantage over the SCs under conditions supportive of SC expansion. These non-glial cells do not require special substrates or purified mitogenic factors, and, as noted here for the first time, they are highly resistant to undergo senescence.

### Cell type selective immunolabeling is required to faithfully identify human SCs and fibroblasts

**Figure 3:**
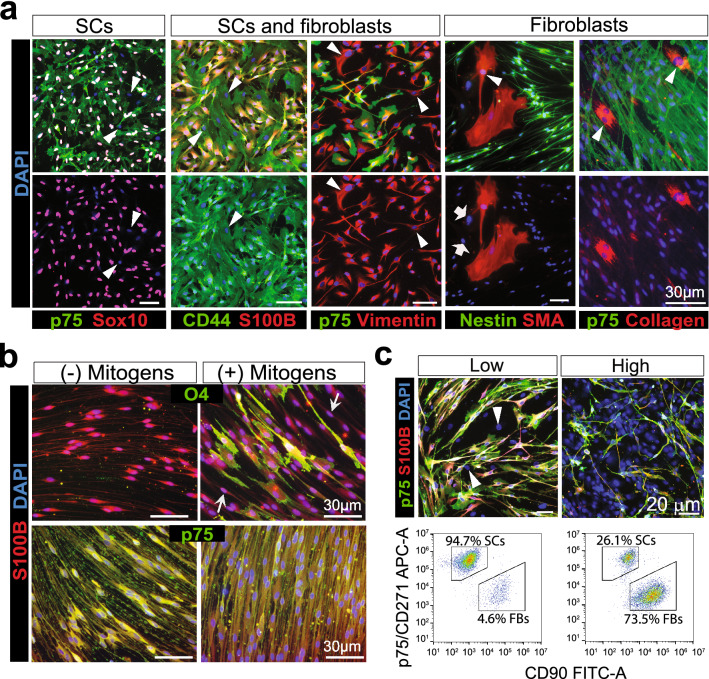
Immunological characterization of mixed populations of human SCs and fibroblasts. (**a**) Useful antibody combinations for co-immunostaining of fixed cells identifying SCs (left), fibroblasts (right) and both cell types (middle panels). Arrowheads in all panels point to selected fibroblasts characterized by the lack of p75^NGFR^, Sox10, S100β and nestin. High levels of vimentin (intracellular, red) and CD44 (membrane, green) were present in SCs and fibroblasts. Most fibroblast cultures contained SMA positive (arrowheads) and negative (arrows) cells. (**b**–**c**) Cell surface immuno-labeling of live human SCs. (**b**) Comparison between O4 and p75^NGFR^ immuno-detection in adherent cells. Sub-confluent cultures of human SCs were incubated for 3 days in SC growth medium (+ mitogens) or in DMEM containing 1% FBS (- mitogens) prior to live cell labeling with O4 and p75^NGFR^ antibodies (green). Immunostaining with S100β antibodies (red) and DAPI (blue) was carried out after fixation. p75^NGFR^ levels were high and homogeneous both in the absence and in the presence of mitogenic factors (**b**, lower panels). By contrast, O4 levels were variable and mitogen-dependent (**b**, upper panels). Some S100β positive SCs did not express O4 (**b**, white arrows) despite prolonged incubation in SC growth medium. (**c**) Fluorescence microscopy imaging (upper panels) and flow cytometry analysis (lower panels) of human cultures affected by various degrees of fibroblast contamination (indicated as high and low in the upper panels). p75^NGFR^ antibodies were used to stain adherent cells (fluorescence microscopy) and cells in suspension (flow cytometry). Thy-1 antibodies (CD90) were used only for flow cytometry analysis due to empirical data showing ineffective detection in adherent (live or fixed) cells. These images attest to the high specificity of S100β (cytoplasmic) and p75^NGFR^ (membrane) co-immunodetection in SCs but not fibroblasts. Whereas Thy-1 (CD90) expression was absent in SCs (**c**), the levels of Thy-1 were heterogeneous in p75^NGFR^ negative cells.

Achieving a thorough understanding of the population used as a starting material is the first step needed to design a successful purification strategy. Generally, SCs are spindle-shaped, elongated cells much smaller in size when compared to fibroblasts, which can be found within clusters of tightly packed cells or intermingling with individual human SCs depending on the experimental setting (Fig. 2a). The appearance of the nuclei can also help discriminate glial from non-glial cells as the nuclei of SCs is roughly oval and more condensed than the one of fibroblasts (Fig. 2a). Yet, cellular morphology can be affected by a variety of factors, including the type of substrate, the confluency of the cultures, and the culture medium. As shown in Fig. 3a and Supplementary Fig. 1, human SCs can adopt a transient flattened, expanded phenotype that resembles the one of fibroblasts and cannot be discriminated clearly unless immunological methods are used.

Thus, we set out to test the reactivity and specificity of a variety of commercially available antibodies with potential to discern SCs from fibroblasts in mixed human nerve-derived cultures. This was needed because most antibodies commonly used to identify rodent (rat) SCs do not cross-react with the respective human antigens or give the same level of specificity. As presented in Fig. 3a, we found a few antibody combinations with capacity to detect cell type-related proteins strongly and specifically in fixed cells. As expected, immunostaining with antibodies against S100β could clearly discern SCs and fibroblasts (Fig. 2a). Alternative antibody combinations involved the detection of the following widely known SC-specific proteins: (1) p75^NGFR^, which is an integral membrane receptor expressed at high levels in SCs and other cells derived from the neural crest (Figs. 2b,3a), (2) nestin, which is a cytoplasmic intermediate filament related to neurofilament expressed in mitotically active nerve cells (Fig. 3a), and (3) Sox10, which is a nuclear transcription factor with a well-assigned role in neural crest development and SC myelination (Fig. 3a). The transcription factors Sox2 and Oct6 exhibited SC-specific nuclear localization, as shown previously^26^, but their uneven levels of expression within the S100β positive population limited their use to discriminate SCs from fibroblasts effectively (not shown). Consistent with a previous report^27^, the intermediate filament vimentin and the anchoring membrane receptor CD44 were evenly expressed in human SCs and fibroblasts (Fig. 3a).

Essentially all S100β positive cells co-expressed CD44 and p75^NGFR^ as determined by image analysis (Supplementary Fig. 1) though some populations displayed nuclear rather than cytoplasmic S100β localization (not shown), as observed previously^26^. For practical purposes, and considering the fact that normal nerve tissues were used to derive our cultures, we have regarded S100β and p75^NGFR^ equally suitable for human SC identification regardless of the cellular localization of the antigenic signal. However, caution should be taken in interpreting S100β immunostaining results in other types of cultures such as those suspected of CNS glia contamination because S100β positive cells that are not SCs are likely to emerge^13,28^.

Unfortunately, we were unable to find a common marker for nerve-resident fibroblasts. Fibronectin was strongly expressed in fibroblasts, but it was also expressed in SCs at the protein (Fig. 2a) and mRNA levels (Fig. 5d). Fibronectin immunostaining is evidenced as a diffuse fibrous network around the cells and this precludes a clear delimitation of positive and negative cells by image analysis (Fig. 2a). In general, we found that detection of ECM proteins such as fibronectin (Fig. 2a), laminin (not shown), and collagen type IV (Fig. 3a) was more reliable when done in conjunction with markers such as S100β and p75^NGFR^. High levels of smooth muscle actin or SMA, a marker typical of myofibroblasts and pericytes, was found in some contaminating cells (Fig. 3a) as reported previously^29^. However, the proportion of SMA positive cells differed in independent cultures and the levels of SMA protein changed drastically with high doses of cAMP (not shown).

Overall, these results indicate the need for cell type-specific immunolabeling in order to unequivocally discern SCs and non-glial cells. This is important considering the morphological variability and the antigenic heterogeneity that lies within SCs and fibroblasts of human origin^13,14^.

### Cell surface p75^NGFR^ immunolabeling is suitable for the quantification and MACS-assisted purification of human SC cultures

Magnetic labeling and separation via MACS require cell type-selective antibodies able to bind to an epitope on the surface of living cells. In addition, the antigen should be stably expressed in the target cells and preserved after trypsinization to allow the labeling of cells in suspension. Two candidate SC-specific membrane antigens were investigated for their potential use in the MACS of human peripheral nerve cells. One of these antigens was p75^NGFR^ and the other was the myelin-specific galactosphingolipid known as O4, which is expressed on the plasma membrane of central and peripheral myelinating glial cells^30^. Antibodies against p75^NGFR^ and O4 are suitable for the MACS of rodent SCs^31,32^. Though cultured human SCs can express O4, the levels are cAMP-dependent and variable within individual cells^33^, as denoted by the rapid disappearance of the O4 signal in cells shifted to forskolin-free medium (Fig. 3b). Indeed, the percentage of O4 positive human SCs rarely surpasses 20% in mitogens medium (Fig. 3b) or SC differentiation medium^14^. By contrast, p75^NGFR^ expression was high and homogenous across all human SC cultures selected for study (Supplementary Fig. 1) irrespective of mitogens and serum (Fig. 3b). p75^NGFR^ antibodies were suitable for live cell labeling under conditions of adherence and suspension, which enabled the quantification of p75^NGFR^ positive cells by fluorescence microscopy imaging and flow cytometry analysis (Fig. 3c). Though MACS of O4 positive human SCs was feasible in certain populations (not shown), the separation of p75^NGFR^ -expressing cells was preferred due to the broad expression of p75^NGFR^ in SCs and its invariance with respect to culture variables, passage number and donor-specific characteristics. Monoclonal antibodies from the hybridoma cell line HB8737 (anti-p75^NGFR^, human-specific) were efficacious to both immuno-stain and magnetically sort SCs in all populations analyzed (Table 1).

**Table 1:** MACS of human SC cultures affected by different degrees of fibroblast growth.

Fibroblasts/SCs	Marker	Positive cells (%)			p value	Recovery (%)	Column type
		Total	Eluted	Retained			
High	p75^NGFR^	20.2 ± 3.9	0.0 ± 0.0	72.1 ± 3.2	< 0.001	76	MS
	S100β	15.5 ± 2.3	0.0 ± 0.0	76.2 ± 4.0	< 0.001		
	p75^NGFR^	27.0 ± 4.6	0.0 ± 0.0	73.7 ± 4.9	< 0.001	75	MS
	S100β	18.1 ± 4.2	0.0 ± 0.0	74.2 ± 5.2	< 0.001		
	p75^NGFR^	70.8 ± 6.7	0.9 ± 0.4	97.6 ± 1.2	< 0.001	51	MS
	S100β	72.6 ± 6.0	0.9 ± 0.4	97.8 ± 1.3	< 0.001		
Moderate	p75^NGFR^	75.1 ± 5.9	1.2 ± 0.7	98.1 ± 0.5	< 0.001	n.d	MS
	S100β	75.3 ± 7.0	1.2 ± 0.7	98.0 ± 0.7	< 0.001		
	p75^NGFR^	82.6 ± 2.3	23.6 ± 2.5	99.5 ± 0.2	< 0.001	100	Large cell
	S100β	78.1 ± 4.0	23.5 ± 4.1	99.6 ± 0.2	< 0.001		
	p75^NGFR^	88.1 ± 3.6	11.5 ± 4.4	98.7 ± 0.5	< 0.001	54	MS
	S100β	87.5 ± 2.1	11.2 ± 4.1	98.7 ± 0.5	< 0.001		
Low	p75^NGFR^	88.9 ± 3.3	3.3 ± 1.7	95.6 ± 0.6	< 0.01	n.d	MS
	S100β	87.8 ± 2.6	3.3 ± 1.7	95.5 ± 0.5	< 0.01		
	p75^NGFR^	93.1 ± 3.1	33.9 ± 5.0	98.8 ± 0.9	< 0.05	91	Large cell
	S100β	90.9 ± 1.9	33.3 ± 4.0	99.9 ± 0.2	< 0.001		

**Figure 4:**
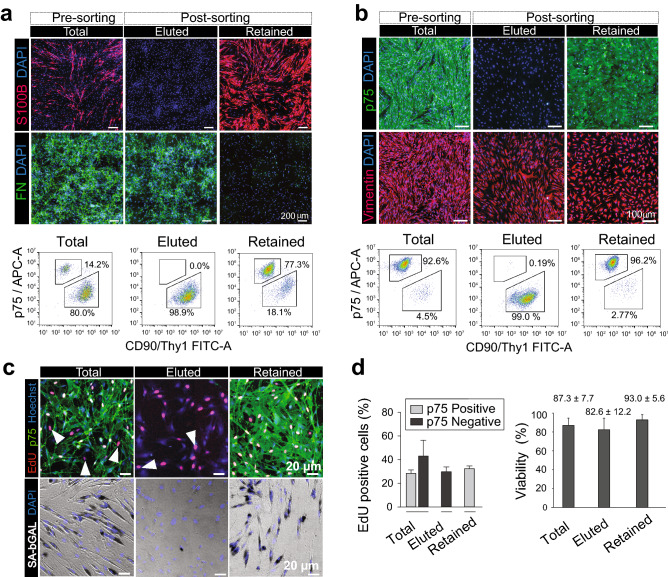
Purification of human SC cultures via MACS. Results from representative experiments using unpurified human SC populations containing high (**a**) and low (**b**–**c**) fibroblast contamination are shown. Cells were labeled in suspension with p75^NGFR^ antibodies and separated using MS-columns (Methods). Samples from the pre-sorting (total) and post-sorting cells were plated in SC growth medium (retained cells) and FBS-only medium (eluted cells) for analysis by immunofluorescence microscopy and flow cytometry. The retained and eluted cells were highly viable (**c**), rapidly adhered to the substrate acquiring the expected pattern of growth (**a**–**c**) and underwent proliferation, as denoted by EdU labeling (red nuclei, **c**). Representative images of a senescent human SC culture (passage-4) subjected to MACS is shown in panel c (bottom images, SA-βGAL activity) to confirm the absence of senescent cells in the eluted fraction. Observe the negligible representation of S100β and p75^NGFR^ positive cells in the populations of eluted cells (**a**–**c**) and the high SC enrichment obtained in one round of purification (**a**). A quantification of the proportion of proliferating (EdU positive) cells discriminated on the basis of p75^NGFR^ expression (green) is shown in panel (**d**) along with an assessment of viability (Trypan blue exclusion assays) for cells in suspension prior to (total) and immediately after MACS (n = 8).

Cellular material from 8 different cultures were subjected to purification via p75^NGFR^ immunolabeling and MACS using two types of columns, as indicated. Experimental conditions were identical to those described in Figs. 4. Passage and donor information for each culture was the following (from top to bottom): passage-2 (17 years old, male); passage-3 (17 years old, male); passage-2 (66 year old, male), passage-2 (18 year old, male); passage-1 (60 year old, male); passage-3 (10 year old, female); passage-3 (60 year old, male); passage-1 (66 year old, male). Quantification of S100β and p75^NGFR^ was carried out by microscopic analysis of serial images obtained by automated fluorescence microscopy. The p-value results from the comparison between samples from total and retained cells (T-test). The viability was determined by Trypan blue exclusion assays using cells in suspension collected immediately after separation. Non-determined measurements are indicated as n.d. Some variability was observed in the percentage of SC enrichment in the retained fraction, as determined primarily by the initial proportion of SCs versus fibroblasts. The recovery of cells (i.e. the ratio between the pre- and post-sorting cell numbers) was variable using MS columns but consistently high using Large Cell columns, which are designed for the separation of cells of a larger diameter.

On a note, we found that negative selection strategies were not entirely effective for human peripheral nerve cultures due to the lack of a membrane antigen expressed universally in the contaminating populations. Despite that rat SC cultures can be depleted of non-glial cells by magnetic separation of Thy-1 positive cells^9^, a similar strategy was not reliable for human SCs (not shown). Thy-1 (also known as CD90) is a cell surface-anchored glycoprotein expressed in fibroblasts rather than SCs^34^. Using fluorescently-tagged CD90 antibodies, we confirmed the expression of the Thy-1 protein in the p75^NGFR^ negative populations (Fig. 3c). However, the finding of atypical human cultures containing p75^NGFR^ negative cells that lacked Thy-1 expression (not shown) discouraged our use of Thy-1 labeling for MACS. Fibronectin labeling was also discouraged based on empirical data showing the inefficacy of monoclonal anti-fibronectin antibodies to separate p75^NGFR^ negative cells (not shown) possibly due to the high levels of fibronectin expressed by SCs (Fig. 2a) or the loss of fibronectin from the cell’s surface after trypsinization. For these reasons, and as shown in the following sections, we pursued a strategy based on positive selection (with p75^NGFR^) rather than negative selection (with Thy-1 or fibronectin) to purify human SCs from mixed cultures. To aid in interpretation, p75^NGFR^ positive, S100β positive cells were referred to as ‘SCs’ whereas p75^NGFR^ negative, S100β negative cells were referred to as ‘fibroblasts’ in the text and figures.

### Optimized MACS protocols allow the fast recovery of highly viable human SCs and fibroblasts

Independent human SC cultures were analyzed for their relative content of p75^NFGR^ positive and negative cells prior to being enzymatically dissociated and separated via MACS. For this, we essentially followed the step-by-step protocol described in our recent paper^11^ but introduced proper adaptations as per the content of fibroblasts to obtain the highest possible yields and purity in the eluted and retained fractions.

Cell batches containing up to 10^7^ magnetically labelled cells were used for MACS. Samples representative of the total (unpurified), retained (enriched in SCs), and eluted fractions (enriched in fibroblasts) were collected and analyzed by immunofluorescence microscopy and flow cytometry in order to determine the efficiency of separation. Cell counts and viability assays were performed concurrently to estimate the recovery of cells in each fraction. Human SC cultures containing fibroblasts at different ratios were selected for optimization and troubleshooting the MACS protocol. Moreover, two types of columns (i.e., MS and Large-cell columns) were tested upon the realization that further adjustments were needed in consideration of the larger size of human SCs and fibroblasts with respect to those of the rat (not shown). The outcome of representative experiments are depicted in Fig. 4 and Table 1. We found that a single round of purification was sufficient to achieve high purity in cultures containing a majority of SCs irrespective of the type of column used (Table 1). The eluted fractions were nearly devoid of SCs with the exception of separations carried out with Large-cell columns, most likely due to the larger pore size of the matrix and higher elution speeds. Though some variability was observed in independent experiments, Large-cell columns were more reliable to maximize SC purity in the retained fractions. Conversely, MS columns were more reliable to minimize SC partitioning into the eluted fraction (Table 1). The purified SCs (retained) and fibroblasts (eluted) were highly viable (Table 1 and Fig. 4d), adhered to the laminin substrate (Fig. 4a–b) and readily responded to mitogenic stimulation by undergoing proliferation, as inferred by their capacity to incorporate EdU (Fig. 4c–d). As shown in Fig. 4c, this method was also appropriate to purify senescent SC populations.

Cultures that contained an excess of fibroblasts over SCs required additional rounds of purification to attain > 95% SC purity (Table 1). Sequential passing of the retained fraction through an additional column (i.e., without introducing an additional labeling step) improved SC purity with the caveat of reduced yield possibly due to cell loss during the procedure (not shown). It is well known that SCs undergo apoptosis when deprived of trophic support or maintained detached for extended periods of time. That is why we implemented changes to minimize the labeling and sorting times and prevent cell death during the procedure. Achieving high purity was possible by performing two sequential rounds of MACS with the introduction of an intermediate step of cell culture (one to two days) to provide prompt adhesion to the retained fraction before attempting a second round of MACS (not shown). The incorporation of 10–20% FBS to the MACS buffer was efficacious to improve cell survival^11^.

In sum, MACS technology provided a unique tool to separate human SCs and fibroblasts in minimal time without compromising the biological activity of the cells for continued culture, direct use in experimentation and storage by cryopreservation, as demonstrated for rat cells^9^.

### Transcriptomics profiling reveals the identity and purity of human SCs and fibroblasts

**Figure 5:**
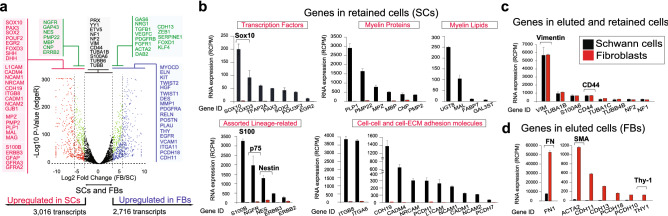
RNA-seq analysis of MACS-purified human SCs and fibroblasts. (**a**) Volcano plot denoting the partitioning of RNA species in SCs and fibroblasts. Genes expressed only in fibroblasts were coded in blue and the ones expressed only in SCs were coded in red. Genes exhibiting > 10–50-fold change in one cell type respect to the other were labeled as ‘predominantly’ expressed in SCs (228 genes, green, left side) or fibroblasts (228 genes, green, right side). Non-differential genes were coded in black (central area). For a reference, genes expressed exclusively in any given cell type included those that exhibited > 2.5 RCPMs in one cell type but no more than 1 RCPM in the other, with at least tenfold change difference in RCPM levels (see Methods). (**b**) Selected protein coding genes known to be associated to the SC phenotype and the myelination program. (**c**) Protein coding genes with high representation in both SCs and fibroblasts. (**d**) Protein coding genes in fibroblasts with low or negligible representation in SCs. In all bar graphs, the bar heights represent the average RCPM value for each identified gene and the error bars represent SD. All genes were called significant (FDR < 0.05) by DESeq2 and edgeR with the exceptions of those displayed in panel (**c**). The partitioning of key SC lineage-specific transcripts in the retained fraction (**b**) attests both to the efficiency of MACS and the sensitivity of the RNA-seq. The cell type-specificity of some mRNAs (brackets) matched our results from immunological analysis.

To achieve the highest possible resolution in gene expression profiling, the eluted and retained cells were processed for RNA isolation and sequenced via RNA-seq. A stringent statistical data analysis indicated that 12,772 transcripts that matched known sequence reads in the *Homo sapiens* reference genome were significantly expressed in SCs and fibroblasts combined. Differentially expressed genes between SCs and fibroblasts encompassed 44.88% of the transcriptome. They consisted of 5,228 coding and 504 non-coding transcripts including long intergenic non-coding RNAs, as highlighted jointly by the algorithms of two different software packages. The volcano plot provides a graphical illustration of the genes expressed exclusively in SCs (445 genes, red dots), exclusively in fibroblasts (441 genes, blue dots) and prevalently in each of these populations (green dots). The transcripts significantly expressed in either SCs or fibroblasts but not considered differential lie within the central area of the plot (black dots). The names of some coding genes in each category were cited in the graphic itself for reference to quantitative data shown in Fig. 5b–d.

Overall, the RNA-seq data validated our immunological strategy. The coding transcripts for the proteins selected for immunostaining, including S100β (S100B), p75^NGFR^ (NGFR), Sox10, nestin (NES), Thy-1/CD90 (THY) and SMA (ACTA2) showed the expected cell type specificity. The mRNA for GFAP, SHH and MAG were detected at low levels (< 4 RCPMs) but specifically in SCs. Vimentin (VIM) and CD44 mRNAs were expressed at equal levels in both fractions (Fig. 5c), also as predicted by data on the immunodetection of the respective proteins.

As expected, the retained fraction displayed genes relevant to the neural crest, the SC phenotype, the control of SC proliferation by neuregulin-ErbB signaling, and the myelin program. Albeit some exceptions, myelin-associated proteins (including the transcriptional enhancer of myelination EGR2 or Krox20) and enzymes involved in myelin lipid biosynthesis were found exclusively in the SC-containing fraction (Fig. 5a,b). It was unexpected to find that the transcripts for the myelin protein periaxin (PRX), the genes associated with neurofibromatosis (NF1, NF2 and ETV5), and the transcriptional regulator YY1 were equally represented in SCs and fibroblasts (Fig. 5a,c), and that Oct7/Brn2 (POU3F2) rather than Oct6 (POU3F1) was the prevalent POU3 isoform in human SCs (Fig. 5b). Other coding genes with high and specific representation in the SC fraction included: (1) membrane receptors of the integrin family (ITGB8 and ITGA6); and (2) cell surface molecules mediating SC/SC and SC/neuron interactions, including cadherin-like proteins (CDH19 and PCDH1), nectin-like proteins (CADM4 and CADM1) and members of the cell adhesion molecule (CAM) family (L1CAM, NRCAM1, and NCAM1/2). In Fig. 5c, the expression of S100A6 and several tubulin isoforms (TUB) are shown as reference for their high and equal distribution between the groups. The distribution of all expressed S100 transcripts is provided in Supplementary Table 1 to illustrate the many S100 variants expressed in SCs and fibroblasts. In Fig. 5d, the most highly expressed cadherin (CDH) isoforms in fibroblasts were selected as examples of genes expressed only in these cells. Although fibronectin (FN1) mRNA was one of the most highly expressed genes in fibroblasts (> 50,000 RCPM), it was also expressed at high levels in SCs (> 7,900 RCPM), (Fig. 5d). Consistent with the well-established contribution of SCs to ECM formation and maintenance^35^, we found that SCs expressed a wide range of isoforms for collagen (COL), laminin (LAM) and ECM remodeling enzymes within the MMP and ADAMs families (not shown). As a reference, the 25 most differentially expressed transcripts with preferential localization in the eluted fraction are provided in Supplementary Table 2.

To summarize, the partitioning of typical mRNA species in each population confirmed the identity of the human SCs and the high purity of the preparations obtained via MACS.

### Gene clustering analysis of transcriptomics data highlights the origin and immaturity of SCs and fibroblasts and the heterogeneity of the fibroblast populations

**Figure 6:**
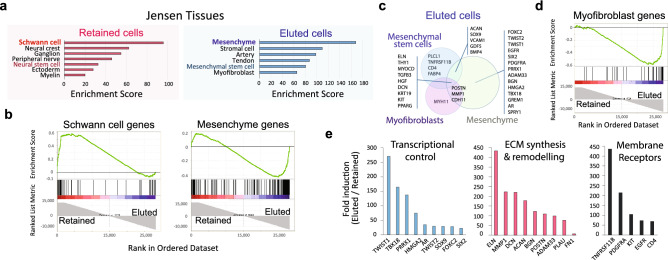
Tissue enrichment analysis of genes expressed in SCs and fibroblasts. The data reflects the output analysis obtained from enrichR using Jensen tissues. The bar graph in (**a**) was adapted directly from the enrichR output data containing the enriched terms (i.e. known cell types most highly represented in the input dataset) arranged in decreasing order of significance according to the combined enrichment score. (**b**) GSEA enrichment plots highlight the enrichment of SC- and mesenchyme-related genes in the retained and eluted fractions, respectively. The GSEA profiles compared the genes in our data set with previously published data on genes expressed in SCs and mesenchymal cells, respectively. In these and all subsequent GSEA plots, the green lines (upper area) indicate the enrichment profile, the vertical black lines (mid area) represent the presence of hits (i.e. a SC- or mesenchymal cell-specific gene in our dataset), and the lower grey areas, represent the metric scores. (**c**) Venn diagram displaying the matching genes for selected terms (mesenchyme, mesenchymal stem cells, and myofibroblasts) in eluted cells to illustrate the gene species accountable for each assignation and their overlap. (**d**) GSEA plot for myofibroblast genes in retained and eluted cells. (**e**) Most highly expressed genes defining the mesenchymal phenotype of fibroblasts organized according to their putative role in transcription, ECM synthesis/remodeling and signaling from membrane receptors.

**Figure 7:**
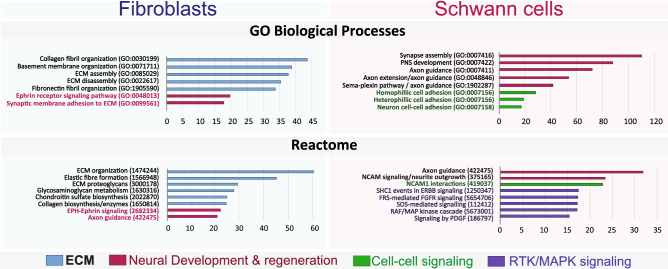
Signal transduction enrichment analysis in human SCs and fibroblasts. The data reflects the output analysis obtained from enrichR using the databases from GO biological processes and Reactome according to the enrichment score (x axis). The bar graphs were adapted directly from the enrichR output display of the indicated enriched terms which comprise the modules of signal transduction that were most highly represented in the input datasets for those genes expressed only in fibroblasts and SCs. As expected, pathway enrichment highlighted genes and networks responsible for collagen synthesis, ECM remodeling (assembly/disassembly) and basal lamina organization in fibroblasts. In SCs, the pathways with the highest scores were those involved in PNS development, maintenance and regeneration as well as cell–cell signaling mediating homophilic and heterophilic interactions with nerve cells. The Reactome database unraveled enrichment in MAPK signaling components in proliferative human SCs. This finding was expected because of the presence of neuregulin in the culture medium^15,16^. Pathways involved in neural regulation in fibroblasts were highlighted by GO biological processes and Reactome.

The list of genes flagged to have unique representation in the retained and eluted fractions were used as data entry in enrichR, a useful software platform for gene clustering and pathway analysis of genome-wide, curated datasets^19^. This allowed us to interrogate the origin and differentiation of SCs and fibroblasts in an unbiased manner in reference to available gene-set libraries of assorted cell types and tissues. The output data from the Jensen Tissues platform highlighted the neural crest (ectodermal) and mesenchymal or stromal origins (mesodermal) of the retained and eluted cells, respectively (Fig. 6a–b). A significant enrichment in genes collectively known for their role in the initiation and maintenance of a stem cell, progenitor state was revealed in both groups (Fig. 6a,c). As such, the human SCs expressed transcripts found during embryonic nerve (PNS) development in early neural crest progenitors (GAP43, FOXD3, TFAP2A, SOX2, SOX10, NGFR, L1CAM, NES, VIM), SC precursors (PLP1, PMP22, CDH19) and immature SCs (PAX3, MPZ, S100B), (Figs. 1 and 6a–b)^36^. Fibroblasts contained genes encoding for stem cell-related receptors and transcriptional regulators such as KIT, TWIST1, TWIST2, FOXC2, PRRX1 and SIX2 (Fig. 6c). The high immaturity of the human SCs was also suggested by their low levels of GFAP (Fig. 5b), the high (> 14 fold) JUN/EGR2 ratio, and the activation of RTK-Ras-MAPK signaling (Fig. 7). Relevant mesenchymal stem cell-associated genes found exclusively in fibroblasts are presented in Fig. 6e with reference to their role in cell signaling, transcriptional control, and ECM synthesis/remodeling. Of particular interest is TWIST1, a basic helix-loop-helix transcription factor that directly activates PRRX1 (also highlighted in our analysis), which is known to maintain the mesenchymal stem cell phenotype as well as the pro-angiogenic and anti-immflamatory function of these cells^37^. TWIST1 and PRRX1 are part of a fibroblast-specific ON/OFF switch for fibroblast activation during wound healing and offer prospect for therapeutic intervention^38^.

Interestingly, the eluted cells contained several transcripts proper of myofibroblasts such as those encoding for ACTA1/SMA , DES/desmin, and the myosin light chain-11 (MYH11), (Fig. 6c–d and Supplementary Table 3), consistent with image data showing the existence of non-glial cells displaying a nestin negative, SMA positive phenotype (Fig. 3a). We found no detectable levels of endothelial- and macrophage-associated genes. However, we cannot exclude the presence of perineurial cells, or cells derived therefrom, despite our efforts to remove as much of the perineurium as possible during the initial dissection of the nerve fascicles and the absence of the typical perineurial gene CLDN1/Claudin-1 (Supplementary Table 3). In fact, the selective partitioning of the newly discovered perineurial marker Jedi-1 [36] in the eluted fraction raises the possibility that perineurial cells contribute to the heterogeneity of the p75^NGFR^ negative populations.

Collectively, our results support the feasibility of using high-resolution genomic methods to unambiguously determine the lineage specificity and the state of maturation of both SCs and fibroblasts.

### Functional analysis of RNAseq data evidences a complex network of interactions between SCs and fibroblasts and their complementary, non-overlapping roles in nerve regeneration

**Figure 8:**
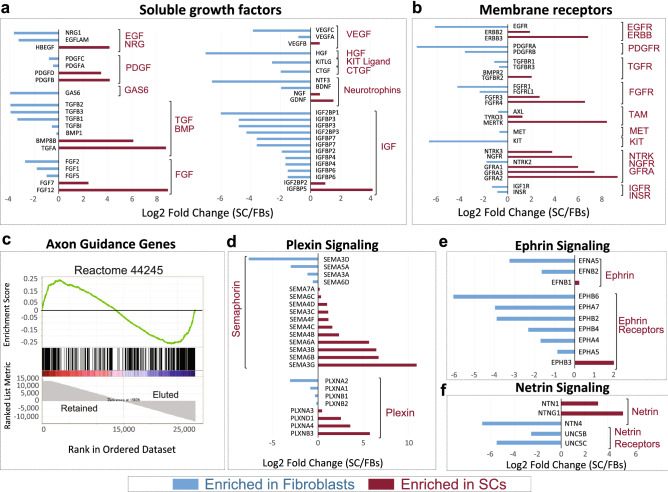
Ligand-receptor profiling in SCs and fibroblasts. (**a**–**b**) Distribution of transcripts encoding for soluble polypeptide growth factors and their cognate plasma membrane receptors. The figure displays selected candidate mediators of SC-fibroblast communication and nerve regeneration as initially highlighted by the output of GO biological processes and Reactome. The most relevant variants/isoforms (> 5 RCPM in SCs or fibroblasts) from each gene family are listed. (**c**–**f**) Distribution of transcripts involved in axon guidance and growth. GSEA enrichment plot as per *Homo sapiens* Reactome ID 44245 (**c**) showing non-overlapping still nearly equal enrichment of axon guidance genes in SCs and fibroblasts. Whereas adhesion molecules (CHL1, NCAM1, NRCAM, L1CAM), ECM molecules (COL9A3, COL9A2), and components of plexin signaling (PLXNB3, SEMA6A, SEMA4D) were highlighted in SCs (**d**), integrin receptors (ITGA10), and components of the ephrin (EPHB6, EPHA7, EPHB2) and netrin signaling pathways (NTN4, UNC5C, ABLIM1) were highlighted in fibroblasts (**e**–**f**). Ligand-receptor pairing was possible for all of the identified classes of ligands (i.e., secreted and membrane-bound ligands) with the exception of VEGF family members (**a**) whose receptors are expected to be present in vascular cells.

We next used enrichR to match our transcriptomics data to functional gene clusters comprising known signal transduction cascades and regulatory elements. The output data from two platforms, namely GO Biological Processes and Reactome, was consistent with the existence of a complex network of putative inter-regulatory relationships among SCs, fibroblasts, neurons and the vasculature. This was in addition to the respective well-recognized roles of SCs and fibroblasts in the development and regeneration of the PNS and the formation and remodeling of a collagen-rich ECM (Fig. 7), respectively. SCs and fibroblasts were well-equipped with a cell type-specific, mostly non-redundant battery of soluble growth factors with potential to act in an autocrine and paracrine fashion in SCs and fibroblasts, as suggested by the cell type-related distribution of the membrane receptors for many of these ligands (Fig. 8a–b).

The fibroblasts expressed various factors known to control SC behavior. One example was the fibroblast-specific expression of the gene encoding for neuregulin-1 (NRG1), which has potential to bind to ErbB2 and ErbB3 receptors in SCs and enhance their proliferation, survival and migration via RTK-Ras-MAPK signaling^15,16^. Another example was the expression of GAS6, a ligand for the TAM receptor tyrosine kinase signaling system, and a known mitogenic factor for human SCs^39^. The expression of the ligand-receptor complexes HGF (hepatocyte growth factor)-MET and KITL (KIT ligand)-KIT in fibroblasts suggests the existence of fibroblast-specific regulatory loops promoting trophic or metabolic support in an autocrine fashion. In addition to this, the fibroblasts expressed various isoforms within the EGF, PDGF, TGF/BMP, FGF, and IGF families (Fig. 8a) with potential to activate receptors expressed in SCs, fibroblasts (Fig. 8b) and other cell types.

The expression of EGF and PDGF isoforms in SCs suggests their capacity to regulate signaling in fibroblasts expressing receptors not found in SCs, such as EGFR and PDGFRA (Figs. 6e and 8b). Hypothetical regulatory networks with neuronal systems and the vasculature can be inferred by the expression of assorted secretable neurotrophic factors and VEGF isoforms, respectively, in SCs and fibroblasts (Fig. 8a). Whereas SCs prevalently expressed the transcripts for GDNF and NGF, fibroblasts expressed the ones for BDNF and NTF3 (Fig. 8a). These ligands have potential to act both in neurons and SCs, as the latter cells expressed high levels of various neurotrophin receptors (Fig. 8b). GFRA members, the receptors for GDNF, were highly and specifically represented in SCs with the following SC/fibroblast partitioning (in RCPM), GFRA1 (1,510 vs 23.9), GFRA2 (619.8 vs 0.95), and GFRA3 (277 vs 1.6).

Surprisingly, axon guidance molecules were enriched in both SCs and fibroblasts; albeit predicted to operate under an independent mechanistic control in each type of cell (Figs. 7 and 8c). Whereas the axon supportive function was linked to NCAM (Fig. 5b) and semaphorin (SEMA)-plexin (PLXN) signaling in SCs (Figs. 7, 8d), it was linked to ephrin (EFN)-ephrin receptor (EPH) and netrin (NTN) signaling in fibroblasts (Figs. 7,8e–f). Curiously, netrin ligands were mostly expressed in SCs and netrin receptors (UNC5 rather than DCC) in fibroblasts (Fig. 8f). The distribution of SEMA-PLXN and EFN-EPH isoforms suggests the possibility of auto- and inter-regulatory signaling involving SCs and fibroblasts.

**Figure 9:**
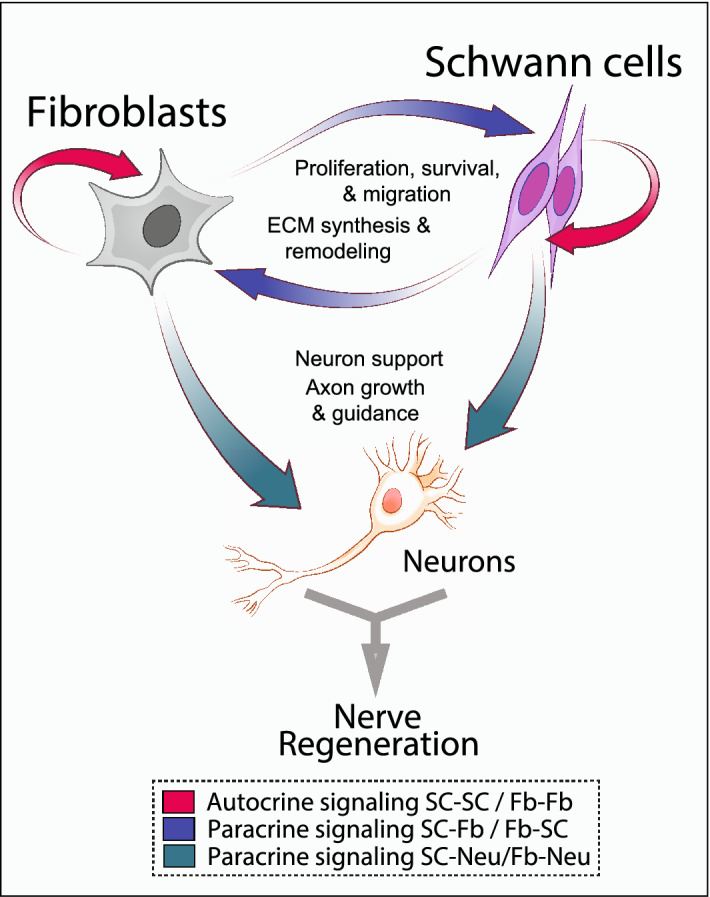
Auto and heterotypic interactions between SCs and fibroblasts in nerve regeneration. The RNA-seq profiling unmasked key hypothetical connections among human SCs and fibroblasts operating via multiple signaling mechanisms. Paracrine signaling from SCs and fibroblasts targeting neurons and/or other cellular components within the nerves, such as the vasculature and immune cells (not shown), can also be anticipated based on RNAseq profiling. Examples of potential molecular players mediating autocrine (red arrows) and paracrine (blue and green arrows) interactions are described in Figs. 7 and 8, and the text.

This data is consistent with the existence of reciprocal regulatory networks between SCs and fibroblasts possibly at play during nerve regeneration (Fig. 9). It seems plausible that SCs and fibroblasts modulate their own function and that of various other cellular components in peripheral nerves via multiple mechanisms of cell–cell communication and ECM remodeling^40^.

## Discussion

In this study, we combined the use of immunofluorescence microscopy analysis, flow cytometry and RNA-seq to unequivocally characterize the prevailing phenotypes developing in primary adult human nerve cultures (Fig. 1). We present useful tools, including effective antibody combinations to detect human antigens, and MACS protocols developed in our lab that enable the highest possible efficiency of separation of the glial cell component while preserving the viability and purity of the non-glial fraction. Our elaborated transcriptomic analysis underscored the independent origin of SCs and fibroblasts, and the complexity of the SC-fibroblast interrelationship at the molecular level (Fig. 9). The novel methods and concepts presented here are relevant to the study of whole nerve tissues^41^, SC-like cells generated from stem cell sources^42^ and neural crest-derived cells including but not restricted to SCs.

### Fibroblast contamination and the need for human-specific methods for analysis and purification

The expansion of the usually small pool of human SCs recovered from a nerve biopsy has been possible since the introduction of the mitogenic combination consisting of neuregulin and forskolin^5,23^. However, these chemical factors do not allow a continuous increase in human SC numbers (as shown for rat SCs), nor do they prevent their purity to be jeopardized by fibroblast overgrowth. Differential adhesion to plastic is a fast and inexpensive method that has been adopted to manufacture clinical grade human SCs because no additional chemicals are introduced to the culture workflow^43^. However, this simple method has value for human SC enrichment rather than fibroblast removal. Whereas complement-mediated cell lysis with Thy-1 antibodies can deplete postnatal and adult rat SCs of contaminating fibroblasts^34,44^ we found this strategy to be ineffective for human SC cultures due to the heterogeneous nature of the contaminating cell types. Instead, MACS could readily and effectively deprive human SCs of contaminant cells, and concomitantly retrieve the unlabeled fraction, in any population containing p75^NGFR^ positive cells. This is an important advantage over purification protocols based on selective or non-selective cell killing or adhesion. In addition, MACS is a clinically relevant tool for therapeutic monitoring, diagnosis and therapy^45^. Magnetic sorting technologies that are similar to the research-grade ones used herein have been approved for clinical use under good manufacturing practices^46^. In addition to the strong cell type selectivity, MACS can isolate rare cells within a population^47^. MACS has allowed the separation of circulating tumor cells for metastatic cancer prognosis^48^ and of endothelial cells for the assessment of cardiovascular injury^49^, thereby making it ideal to possibly isolate SC-like cells from tumors and other unconventional locations.

The early detection and elimination of non-glial cells is essential for any intended use of the human SC cultures but it is particularly relevant in SC auto-transplantation therapy^10,50^. Although they were first described as cells of mesodermal origin, fibroblasts are known to exist in different populations. Some fibroblasts derive from the neural crest and have an ectodermal origin^51^. The diversity and heterogeneity of fibroblasts thwarts the finding of a general fibroblast-specific marker. We found that human nerve-derived fibroblasts are an heterogeneous, non-senescent population different from rodent fibroblasts which are known to acquire senescence in vitro^52^. Some fundamental differences in the expression of certain genes became apparent between human peripheral nerve fibroblasts that are PAX3 negative, ETV5 positive (our study) and skin-derived fibroblasts from the human dermis that are PAX3 positive, ETV5 negative as described by Stratton et al.^26^. Most strikingly, peripheral nerve fibroblasts displayed a strong neural signature as judged by their high levels of expression of genes responsible for the development and regeneration of neural tissues. In fact, a general understanding is that the phenotype of fibroblasts is varied and may be dependent on the species in question and the tissue of origin^53^.

Even though we have defined the eluted cells collectively as ‘fibroblasts’, we understand that they are an undefined and variable group of cells whose origin is still uncertain. We assume that the majority of the p75^NGFR^ negative cells derive from endoneurial fibroblasts but the expression of SMA suggests the presence of pericytes^29^. It is possible that the SMA positive cells found in our human nerve cultures are analogous to the mouse nerve pericytes described by Stierli et al.^54^ as p75^NGFR^ negative, NG2 positive, PDGFRβ positive, ACTA/SMA positive cells^54^. Yet, the eluted cells do not seem to contain endoneurial fibroblasts analogous to the ones reported in the same study (defined as p75^NGFR^ positive, S100β negative, NG2 positive, PDGFRβ positive, SMA negative cells) mainly because of the lack of S100β negative, p75^NGFR^ positive cells our typical preparations. Nevertheless, it is possible that our cultures contain perineurial cells that have lost typical cell adhesion markers such as Claudin-1. Whether different nerves render cultures with different characteristics or levels of purity is unknown, as many sources of variability can affect the quality and quantity of the human SC cultures^13^. The heterogeneity of the populations may reflect the influence of extracellular factors in gene expression profiles or an unintended selection of the most aggressive phenotypes. Yet, we assume that basic characteristics, such as the expression of lineage-specific genes, are rather constant and not artefactual from the culture conditions^13,28^. To this end, more studies are needed to trace the SCs and non-glial cells from the nerve to the culture. A more elaborated analysis of the constitution of the human SC cultures via single cell RNA-seq analysis and/or further purification of existing phenotypes could be particularly enlightening regarding cell type identification.

### The power of transcriptomics to unravel novel features of human SCs and fibroblasts

The availability of the whole transcriptome has enabled us to rigorously compare human SCs and fibroblasts as well as to establish unbiased links to phenotypic and functional states on the basis of existent high-resolution datasets. Our characterization of human SCs was, for the most part, consistent with published data on human SC cultures from nerve^14,27^ and skin^26^. Yet, our analysis revealed a myriad of differentially expressed transcripts with prevalent representation in SCs and fibroblasts that offer potential to be considered as cell type-specific identifiers or be further investigated for their role in human nerve physiology or pathology. Curiously, the fibroblasts displayed a mesenchymal stem cell phenotype. This is consistent with previous findings showing that fibroblasts within the connective tissue sheaths of avian peripheral nerves derive from the mesenchyme rather than the neural crest^55^, that Thy-1 positive cells from rat nerves give rise to perineurium in vitro^56^, and that multipotent mesenchymal precursor cells from adult nerves contribute to skin repair and digit tip regeneration^57^.

One shared property between SCs and fibroblasts was their marked profile of undifferentiated, progenitor-like cells with concomitant expression of assorted, yet mostly non-overlapping, neural-related genes. The relative immaturity of cultured human SCs has been previously recognized by functional studies of neuron-glia interactions^14^ and their immunological profile consistent with the repair SC phenotype^27^. New studies in mice have shown that the wound microenvironment can drive SCs to acquire an invasive, mesenchymal-like state in vivo^58^ but it seems possible that some traits acquired in response to axon-deprivation (or injury induction) are also maintained or recapitulated in vitro. It is hard to ascertain the origin of the human SCs by examination of their molecular characteristics in vitro. This was clearly demonstrated by Stratton et al. who reported that skin-derived and nerve-derived cultured human SCs are transcriptionally undistinguishable^26^. We have found that extended passaging affects the expression of certain genes involved in cell cycle control but we have not seen changes in the levels of S100β, p75^NGFR^ or any of the markers used here for SC identification. The SC transcriptome is remarkably stable with subculture^59^ but whether this is the case for fibroblasts is unknown.

The immaturity of fibroblasts was by far intriguing but was consistent with their high proliferative capacity and lack of replicative senescence. Our functional analysis of the transcriptome suggests that the fibroblasts provide crucial mitogenic and survival factors to human SCs, including neuregulin and GAS6. A role for SCs in modulating aspects of fibroblast physiology via membrane-membrane signaling and soluble ligands was also revealed. The cell type-specific distribution of ligands and receptors involved in cell–cell interactions via homophilic and heterophilic plasma membrane adhesion molecules, as well as the profile of secreted factors (including neurotrophic factors) was consistent with current knowledge regarding the interplay between SCs and fibroblasts in controlling nerve regeneration. Ephrin, netrin and plexin were three major players identified to possibly mediate SC-fibroblast interactions or interactions with neuronal cells supporting axon growth and guidance. Our findings are in agreement with previous studies showing that EphrinB/EphB2 signaling between fibroblasts and SCs directs the coordinated migration of SCs to guide regenerating axons across the bridge^8^ and that Netrin-1 secreted by SCs interacts with DCC receptors on axons to direct their growth^60,61^. Recent research has shown that Ephrin signaling plays a role during SC invasion of CNS lesions^62^. How the plexin pathway regulates PNS regeneration is poorly understood but the high representation and diversity of SEMA and PLXN isoforms in both SCs and fibroblasts suggests the importance of this pathway in mediating bidirectional SC-to-fibroblast communication.

### Concluding remarks

The unique potential of SCs for remyelination along with their provision of an extracellular environment supportive of neural regeneration have converted these glial cells into excellent candidates for transplantation in the PNS and CNS when used as purified cells or tissue grafts^41,50^. The traditional view that fibroblasts contribute scar tissue and negatively affect the outcome of SC transplantation is changing upon new evidence indicating that they assist in tissue regeneration. Fibroblasts promote neurite outgrowth and secrete growth factors and ECM proteins able to stimulate SC migration^40,63,64^. The expression of neurotrophic factors and adhesion molecules supportive of axon growth and pathfinding in fibroblasts further sustain this emerging view. Indeed, it seems that reciprocal signaling between SCs and fibroblasts contribute to cell proliferation, migration, ECM remodeling, and communication with other nerve-resident cell types. Exploiting the pro-regenerative properties of PNS-derived fibroblasts, alone or in combination with SCs, may be considered to optimize therapeutic approaches or evaluate the outcome of preclinical and clinical studies of cell transplants.

Altogether, our studies showed that MACS technology can readily separate human SCs and fibroblasts with retention of biological activity in both populations. Not only are these methods versatile but also lend themselves suitable to the manufacture of clinical grade human SCs for diagnosis or grafting. The transcriptome is a valuable reference tool for cell phenotyping and comparisons of RNA expression profiles in single cells and whole tissues deriving from humans. This tool is ideal for elaborating new mechanistic hypotheses and searching for candidate molecules with potential use in selective cell targeting, in vitro modeling of disease states, and regenerative therapies.

## Supplementary information

Supplementary legends

Supplementary table1

Supplementary table2

Supplementary table3

Supplementary figure1

## Data Availability

Additional data and materials will be made available upon request to the corresponding author.
