# Supplementary material for: Magnetic separation of peripheral nerve-resident cells underscores key molecular features of human Schwann cells and fibroblasts: an immunochemical and transcriptomics approach
Source: Sci Rep. 2020 Oct 28;10:18433. doi: 10.1038/s41598-020-74128-3 (PMC7595160; doi:10.1038/s41598-020-74128-3)
Supplement: Supplementary file 1 — Supplementary legends [file 41598_2020_74128_MOESM1_ESM.docx]

**Supplementary Figures and Tables**

**Supplementary Table 1**. RNA-seq analysis of S100 genes in human SCs and fibroblasts. The table includes all S100A and S100B isoforms highlighted to be expressed in eluted and/or retained cells. The levels of expression of S100 genes are shown in comparison to three typical SC-specific genes (SOX10, NGFR and MPZ).

**Supplementary Table 2**. RNA-seq analysis of the most differentially expressed transcripts in fibroblast cells. The table includes output data from coding transcripts with exclusive or prevalent representation in eluted cells. The top 25 transcripts with expression levels >15 RCPM were selected for display.

**Supplementary Table 3**. RNA-seq analysis of genes known to be expressed in potentially contaminating phenotypes. The table includes output data from selected genes coding for proteins commonly used as markers for the identification of endothelial cells, macrophages, perineurial cells and pericytes, as indicated. The levels of expression are shown in RCPM. Genes expressed at <2.5 RCPM were considered ‘not expressed’.

**Supplementary Figure 1**. Colocalization of S100β and CD44 (a) and S100β and p75^NGFR^ (b) in purified human SCs. Early passage SC cultures were purified by MACS and stained with antibodies against S100β (red) and CD44 or p75^NGFR^ (green) 2 days after separation. Virtually all S100β positive cells (cytoplasmic staining) showed membrane staining for CD44 (a) and p75^NGFR^ (b). No atypical cells were identified in the cultures selected for purification and analysis. Representative areas of purified SCs (retained cells) from 3 different donors are shown in (b). Donor-1 (17 year-old, male), Donor-2 (51 year-old, male); Donor-3 (60 year-old, male). Arrowheads in A denote proliferating SCs.
